# Supplementary material for: Resolving the full spectrum of human genome variation using Linked-Reads
Source: Genome Res. 2019 Apr;29(4):635–45. doi: 10.1101/gr.234443.118 (PMC6442396; doi:10.1101/gr.234443.118)
Supplement: Supplemental Material [file supp_gr.234443.118_Supplemental_longranger-2.2.zip › longranger-2.2.tar.bz2!/lr2.2-public/lib/stan/cmdstan/stan/doc/api/html/classstan_1_1io_1_1var__context.html]

Stan: stan::io::var\_context Class Reference


|  |  |
| --- | --- |
|  | Stan  2.5.0  probability, sampling & optimization |


- Main Page
- Related Pages
- Namespaces
- Classes
- Files

- Class List
- Class Index
- Class Hierarchy
- Class Members

All Classes Namespaces Files Functions Variables Typedefs Enumerations Enumerator Friends Macros Pages

- stan
- io
- var\_context

Public Member Functions |
Static Public Member Functions |
List of all members

stan::io::var\_context Class Referenceabstract

A `var_reader` reads array variables of integer and floating point type by name and dimension.
More...

`#include <var_context.hpp>`

Inheritance diagram for stan::io::var\_context:

|  |  |
| --- | --- |
| Public Member Functions | |
| virtual | ~var\_context () |
|  | |
| virtual bool | contains\_r (const std::string &name) const =0 |
|  | Return `true` if the specified variable name is defined. More... |
|  | |
| virtual std::vector< double > | vals\_r (const std::string &name) const =0 |
|  | Return the floating point values for the variable of the specified variable name in last-index-major order. More... |
|  | |
| virtual std::vector< size\_t > | dims\_r (const std::string &name) const =0 |
|  | Return the dimensions for the specified floating point variable. More... |
|  | |
| virtual bool | contains\_i (const std::string &name) const =0 |
|  | Return `true` if the specified variable name has integer values. More... |
|  | |
| virtual std::vector< int > | vals\_i (const std::string &name) const =0 |
|  | Return the integer values for the variable of the specified name in last-index-major order or the empty sequence if the variable is not defined. More... |
|  | |
| virtual std::vector< size\_t > | dims\_i (const std::string &name) const =0 |
|  | Return the dimensions of the specified floating point variable. More... |
|  | |
| virtual void | names\_r (std::vector< std::string > &names) const =0 |
|  | Return a list of the names of the floating point variables in the context. More... |
|  | |
| virtual void | names\_i (std::vector< std::string > &names) const =0 |
|  | Return a list of the names of the integer variables in the context. More... |
|  | |
| void | add\_vec (std::stringstream &msg, const std::vector< size\_t > &dims) const |
|  | |
| void | validate\_dims (const std::string &stage, const std::string &name, const std::string &base\_type, const std::vector< size\_t > &dims\_declared) const |
|  | |

|  |  |
| --- | --- |
| Static Public Member Functions | |
| static std::vector< size\_t > | to\_vec () |
|  | |
| static std::vector< size\_t > | to\_vec (size\_t n1) |
|  | |
| static std::vector< size\_t > | to\_vec (size\_t n1, size\_t n2) |
|  | |
| static std::vector< size\_t > | to\_vec (size\_t n1, size\_t n2, size\_t n3) |
|  | |
| static std::vector< size\_t > | to\_vec (size\_t n1, size\_t n2, size\_t n3, size\_t n4) |
|  | |
| static std::vector< size\_t > | to\_vec (size\_t n1, size\_t n2, size\_t n3, size\_t n4, size\_t n5) |
|  | |
| static std::vector< size\_t > | to\_vec (size\_t n1, size\_t n2, size\_t n3, size\_t n4, size\_t n5, size\_t n6) |
|  | |
| static std::vector< size\_t > | to\_vec (size\_t n1, size\_t n2, size\_t n3, size\_t n4, size\_t n5, size\_t n6, size\_t n7) |
|  | |
| static std::vector< size\_t > | to\_vec (size\_t n1, size\_t n2, size\_t n3, size\_t n4, size\_t n5, size\_t n6, size\_t n7, size\_t n8) |
|  | |

## Detailed Description

A `var_reader` reads array variables of integer and floating point type by name and dimension.

An array's dimensionality is described by a sequence of (unsigned) integers. For instance, `(7, 2, 3)` picks out a 7 by 2 by 3 array. The empty dimensionality sequence `()` is used for scalars.

Multidimensional arrays are stored in column-major order, meaning the first index changes the most quickly.

If a variable has integer variables, it should return those integer values cast to floating point values when accessed through the floating-point methods.

Definition at line 29 of file var\_context.hpp.

## Constructor & Destructor Documentation

|  |  |  |  |  |
| --- | --- | --- | --- | --- |
| virtual stan::io::var\_context::~var\_context | ( |  | ) |  |

inlinevirtual

Definition at line 31 of file var\_context.hpp.

## Member Function Documentation

|  |  |  |  |
| --- | --- | --- | --- |
| void stan::io::var\_context::add\_vec | ( | std::stringstream & | *msg*, |
|  |  | const std::vector< size\_t > & | *dims* |
|  | ) |  | const |

inline

Definition at line 114 of file var\_context.hpp.

|  |  |  |  |  |  |
| --- | --- | --- | --- | --- | --- |
| virtual bool stan::io::var\_context::contains\_i | ( | const std::string & | *name* | ) | const |

pure virtual

Return `true` if the specified variable name has integer values.

Parameters
:   |  |  |
    | --- | --- |
    | name | Name of variable. |

Returns
:   `true` if an integer variable of the specified name is defined.

Implemented in stan::io::dump, and stan::json::json\_data.

|  |  |  |  |  |  |
| --- | --- | --- | --- | --- | --- |
| virtual bool stan::io::var\_context::contains\_r | ( | const std::string & | *name* | ) | const |

pure virtual

Return `true` if the specified variable name is defined.

This method should return `true` even if the values are all integers.

Parameters
:   |  |  |
    | --- | --- |
    | name | Name of variable. |

Returns
:   `true` if the variable exists with real values.

Implemented in stan::io::dump, and stan::json::json\_data.

|  |  |  |  |  |  |
| --- | --- | --- | --- | --- | --- |
| virtual std::vector<size\_t> stan::io::var\_context::dims\_i | ( | const std::string & | *name* | ) | const |

pure virtual

Return the dimensions of the specified floating point variable.

If the variable doesn't exist (or if it is a scalar), the return result should be the empty vector.

Parameters
:   |  |  |
    | --- | --- |
    | name | Name of variable. |

Returns
:   Sequence of dimensions for the variable.

Implemented in stan::io::dump, and stan::json::json\_data.

|  |  |  |  |  |  |
| --- | --- | --- | --- | --- | --- |
| virtual std::vector<size\_t> stan::io::var\_context::dims\_r | ( | const std::string & | *name* | ) | const |

pure virtual

Return the dimensions for the specified floating point variable.

If the variable doesn't exist or if it is a scalar, the return result should be the empty vector.

Parameters
:   |  |  |
    | --- | --- |
    | name | Name of variable. |

Returns
:   Sequence of dimensions for the variable.

Implemented in stan::io::dump, and stan::json::json\_data.

|  |  |  |  |  |  |
| --- | --- | --- | --- | --- | --- |
| virtual void stan::io::var\_context::names\_i | ( | std::vector< std::string > & | *names* | ) | const |

pure virtual

Return a list of the names of the integer variables in the context.

Parameters
:   |  |  |
    | --- | --- |
    | names | Vector to store the list of names in. |

Implemented in stan::io::dump, and stan::json::json\_data.

|  |  |  |  |  |  |
| --- | --- | --- | --- | --- | --- |
| virtual void stan::io::var\_context::names\_r | ( | std::vector< std::string > & | *names* | ) | const |

pure virtual

Return a list of the names of the floating point variables in the context.

Parameters
:   |  |  |
    | --- | --- |
    | names | Vector to store the list of names in. |

Implemented in stan::io::dump, and stan::json::json\_data.

|  |  |  |  |  |
| --- | --- | --- | --- | --- |
| static std::vector<size\_t> stan::io::var\_context::to\_vec | ( |  | ) |  |

inlinestatic

Definition at line 179 of file var\_context.hpp.

|  |  |  |  |  |  |
| --- | --- | --- | --- | --- | --- |
| static std::vector<size\_t> stan::io::var\_context::to\_vec | ( | size\_t | *n1* | ) |  |

inlinestatic

Definition at line 182 of file var\_context.hpp.

|  |  |  |  |
| --- | --- | --- | --- |
| static std::vector<size\_t> stan::io::var\_context::to\_vec | ( | size\_t | *n1*, |
|  |  | size\_t | *n2* |
|  | ) |  |  |

inlinestatic

Definition at line 187 of file var\_context.hpp.

|  |  |  |  |
| --- | --- | --- | --- |
| static std::vector<size\_t> stan::io::var\_context::to\_vec | ( | size\_t | *n1*, |
|  |  | size\_t | *n2*, |
|  |  | size\_t | *n3* |
|  | ) |  |  |

inlinestatic

Definition at line 193 of file var\_context.hpp.

|  |  |  |  |
| --- | --- | --- | --- |
| static std::vector<size\_t> stan::io::var\_context::to\_vec | ( | size\_t | *n1*, |
|  |  | size\_t | *n2*, |
|  |  | size\_t | *n3*, |
|  |  | size\_t | *n4* |
|  | ) |  |  |

inlinestatic

Definition at line 201 of file var\_context.hpp.

|  |  |  |  |
| --- | --- | --- | --- |
| static std::vector<size\_t> stan::io::var\_context::to\_vec | ( | size\_t | *n1*, |
|  |  | size\_t | *n2*, |
|  |  | size\_t | *n3*, |
|  |  | size\_t | *n4*, |
|  |  | size\_t | *n5* |
|  | ) |  |  |

inlinestatic

Definition at line 210 of file var\_context.hpp.

|  |  |  |  |
| --- | --- | --- | --- |
| static std::vector<size\_t> stan::io::var\_context::to\_vec | ( | size\_t | *n1*, |
|  |  | size\_t | *n2*, |
|  |  | size\_t | *n3*, |
|  |  | size\_t | *n4*, |
|  |  | size\_t | *n5*, |
|  |  | size\_t | *n6* |
|  | ) |  |  |

inlinestatic

Definition at line 221 of file var\_context.hpp.

|  |  |  |  |
| --- | --- | --- | --- |
| static std::vector<size\_t> stan::io::var\_context::to\_vec | ( | size\_t | *n1*, |
|  |  | size\_t | *n2*, |
|  |  | size\_t | *n3*, |
|  |  | size\_t | *n4*, |
|  |  | size\_t | *n5*, |
|  |  | size\_t | *n6*, |
|  |  | size\_t | *n7* |
|  | ) |  |  |

inlinestatic

Definition at line 233 of file var\_context.hpp.

|  |  |  |  |
| --- | --- | --- | --- |
| static std::vector<size\_t> stan::io::var\_context::to\_vec | ( | size\_t | *n1*, |
|  |  | size\_t | *n2*, |
|  |  | size\_t | *n3*, |
|  |  | size\_t | *n4*, |
|  |  | size\_t | *n5*, |
|  |  | size\_t | *n6*, |
|  |  | size\_t | *n7*, |
|  |  | size\_t | *n8* |
|  | ) |  |  |

inlinestatic

Definition at line 247 of file var\_context.hpp.

|  |  |  |  |
| --- | --- | --- | --- |
| void stan::io::var\_context::validate\_dims | ( | const std::string & | *stage*, |
|  |  | const std::string & | *name*, |
|  |  | const std::string & | *base\_type*, |
|  |  | const std::vector< size\_t > & | *dims\_declared* |
|  | ) |  | const |

inline

Definition at line 124 of file var\_context.hpp.

|  |  |  |  |  |  |
| --- | --- | --- | --- | --- | --- |
| virtual std::vector<int> stan::io::var\_context::vals\_i | ( | const std::string & | *name* | ) | const |

pure virtual

Return the integer values for the variable of the specified name in last-index-major order or the empty sequence if the variable is not defined.

Parameters
:   |  |  |
    | --- | --- |
    | name | Name of variable. |

Returns
:   Sequence of integer values.

Implemented in stan::io::dump, and stan::json::json\_data.

|  |  |  |  |  |  |
| --- | --- | --- | --- | --- | --- |
| virtual std::vector<double> stan::io::var\_context::vals\_r | ( | const std::string & | *name* | ) | const |

pure virtual

Return the floating point values for the variable of the specified variable name in last-index-major order.

This method should cast integers to floating point values if the values of the named variable are all integers.

If there is no variable of the specified name, the empty vector is returned.

Parameters
:   |  |  |
    | --- | --- |
    | name | Name of variable. |

Returns
:   Sequence of values for the named variable.

Implemented in stan::io::dump, and stan::json::json\_data.

---

The documentation for this class was generated from the following file:

- src/stan/io/var\_context.hpp


---

[ Stan Home Page ]

*© 2011–2014,
Stan Development Team.*
